# Zika virus infection causes temporary paralysis in adult mice with motor neuron synaptic retraction and evidence for proximal peripheral neuropathy

**DOI:** 10.1038/s41598-019-55717-3

**Published:** 2019-12-20

**Authors:** John D. Morrey, Alexandre L. R. Oliveira, Hong Wang, Katherine Zukor, Mateus Vidigal de Castro, Venkatraman Siddharthan

**Affiliations:** 1 Institute for Antiviral Research, Department of Animal, Dairy, and Veterinary Sciences, 5600 Old Main Hill, Utah State University, Logan, Utah 84322-5600 United States of America; 2 Institute of Biology, University of Campinas, Campinas, SP Brazil

**Keywords:** Virology, Spinal cord

## Abstract

Clinical evidence is mounting that Zika virus can contribute to Guillain-Barré syndrome which causes temporary paralysis, yet the mechanism is unknown. We investigated the mechanism of temporary acute flaccid paralysis caused by Zika virus infection in aged interferon αβ-receptor knockout mice used for their susceptibility to infection. Twenty-five to thirty-five percent of mice infected subcutaneously with Zika virus developed motor deficits including acute flaccid paralysis that peaked 8-10 days after viral challenge. These mice recovered within a week. Despite Zika virus infection in the spinal cord, motor neurons were not destroyed. We examined ultrastructures of motor neurons and synapses by transmission electron microscopy. The percent coverage of motor neurons by boutons was reduced by 20%; more specifically, flattened-vesicle boutons were reduced by 46%, and were normalized in recovering mice. Using electromyographic procedures employed in people to help diagnose Guillain-Barré syndrome, we determined that nerve conduction velocities between the sciatic notch and the gastrocnemius muscle were unchanged in paralyzed mice. However, F-wave latencies were increased in paralyzed mice, which suggests that neuropathy may exist between the sciatic notch to the nerve rootlets. Reversible synaptic retraction may be a previously unrecognized cofactor along with peripheral neuropathy for the development of Guillain-Barré syndrome during Zika virus outbreaks.

## Introduction

Congenital Zika virus (ZIKV) syndrome and Guillain-Barré syndrome are two serious outcomes associated with ZIKV outbreaks^1–3^. Guillain-Barré syndrome is a reversible, acute peripheral neuropathy. It involves varying levels of limb or cranial muscle weakness, diminished deep tendon reflexes, and albuminocytologic disassociation (elevated protein levels with normal cell counts in cerebrospinal fluid) characteristic of Guillain-Barré syndrome^4,5^. Fifty to seventy-five percent of Guillain-Barré syndrome cases occur within a couple of weeks after a respiratory or gastrointestinal infection or perhaps other immune stimuli that triggers autoimmune responses affecting the peripheral nerves and spinal roots^6^.

ZIKV has not been proven to cause Guillain-Barré syndrome^7^; however, the following multiple epidemiological observations suggest that ZIKV may contribute to Guillain-Barré syndrome. ZIKV outbreaks coincide with increased incidence of Guillain-Barré syndrome. When ZIKV outbreaks cease, the disease incidence declines. ZIKV infection has also been identified in cases of Guillain-Barré syndrome^8^. Moreover, modeling of epidemiological data from 11 locations which report cases of both ZIKV and Guillain-Barré syndrome indicate that the incidence of Guillain-Barré syndrome during a ZIKV outbreak can be many times higher than normal^8^.

Rodent models of Guillain-Barré syndrome, not infected with ZIKV, have been valuable for understanding the human disease, but they do not model all aspects of human Guillain-Barré syndrome. The most widely used animal model, experimental allergic neuritis (EAN), is induced experimentally by immunization of peripheral nerves or associated proteins^9,10^, or by adoptive transfer of sensitized T-cells to such proteins^11,12^. Spontaneous Guillain-Barré syndrome-like models in mice have furthered our understanding of Guillain-Barré syndrome^13,14^, but questions still remain regarding many other unknown aspects of human Guillain-Barré syndrome.

It is not unreasonable to expect that animal models, such as the one described herein, could model some aspects of human Guillain-Barré syndrome disease. As in other examples, ZIKV replication of dorsal root ganglia explants from *IFNAR*^−/−^ mice undergo demyelination^15^. In another study, C1q-specific antibodies were detected in the sera of interferon αβγ-receptor knockout (AG129) mice infected with ZIKV^16^. The important point is that innovative approaches can advance the understanding of human disease despite the lack of perfect animal models.

In this report, we describe a unique mouse model of ZIKV-induced acute flaccid paralysis where mice recover from paralysis within a week. Unlike most other models of flavivirus encephalomyelitis^17,18^, motor neurons do not die in this ZIKV-induced paralysis model. We chose to investigate synaptic retraction, because retraction of pre-synaptic terminals from spinal motor neuron (MN) cell bodies has been associated with acute flaccid paralysis in mouse and rat experimental autoimmune encephalomyelitis models of relapsing multiple sclerosis^19,20^. Most compelling is that synapses are retracted in paralyzed animals and are re-connected with MNs days later when symptoms are abated.

Electromyographic data suggested proximal neuropathy occurs in this ZIKV-induced acute flaccid paralysis model. A mechanism involving synaptic retraction as a co-factor with peripheral neuropathy not involving MN death will help in our understanding of flavivirus-induced paralysis and perhaps ZIKV-induced Guillain-Barré syndrome.

## Results

### Quantitative measurement of ZIKV-induced paralysis

**Figure 1:**
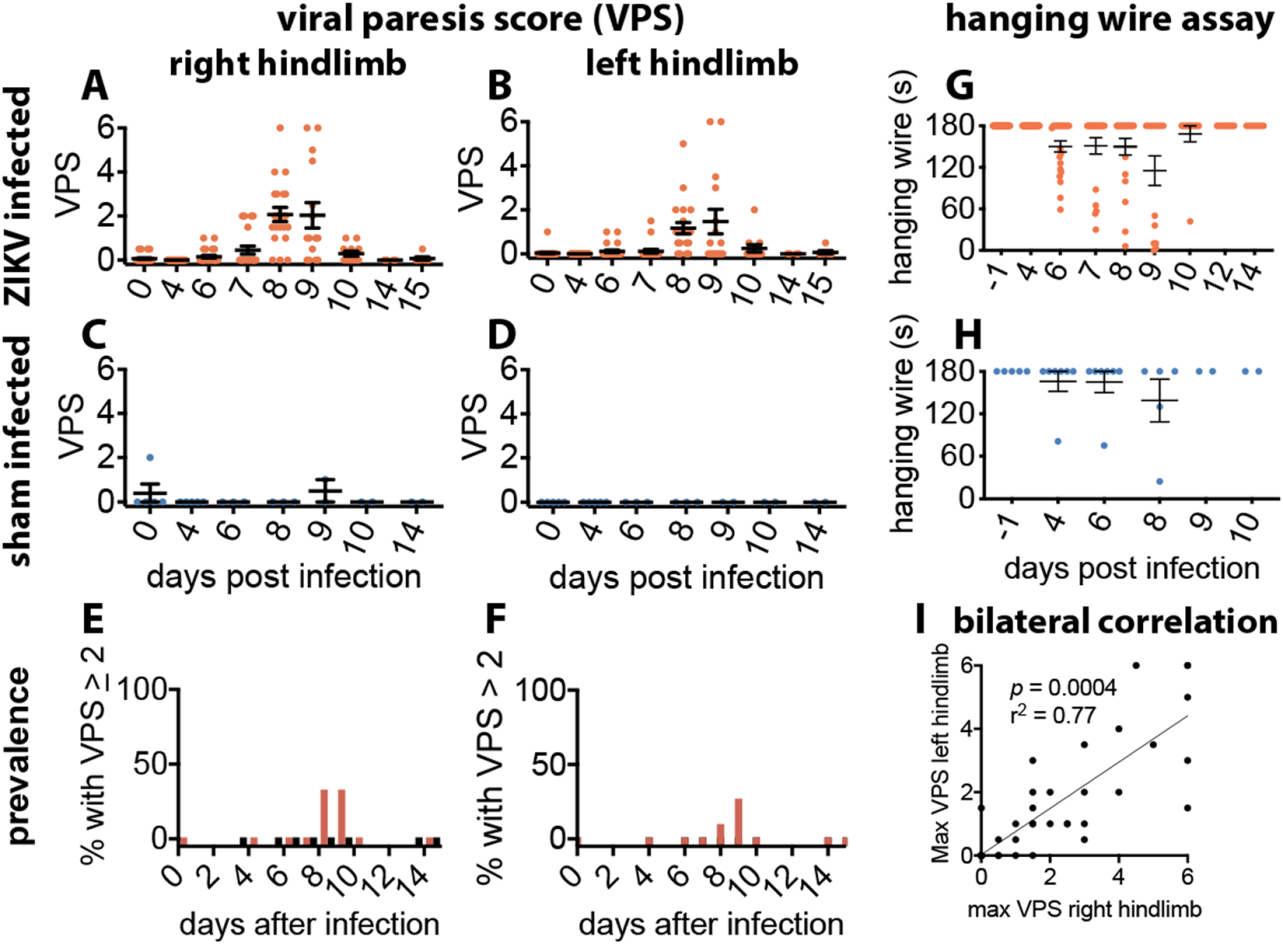
Motor deficits and acute flaccid paralysis in ZIKV-infected 5.0- to 5.3-month-old *IFNAR*^−/−^ mice (male = 20, females = 18). Viral paresis scale (VPS) was used to monitor motor deficits through day 15 after subcutaneous viral challenge (6.7 × 10^3^ pfu/mouse) in the right-side inguinal area. Dots represent data points for each right and left hindlimb (**A**–**D, G**–**I**), or mean group values (**E**,**F**). (**A**,**B**) ZIKV-infected mice (orange), and (**C,D**) sham-infected mice (blue). Prevalence of motor deficits was identified as percent of animals with viral paresis scale >2 on the (**E**) right hindlimb and (**F**) left hindlimb. Hanging wire test of (**G**) ZIKV- and (**H**) sham-infected mice. (**I**) Correlation of right and left hindlimb viral paresis scale substantiates bilateral motor deficits. Mice were depleted over time from necropsies at day 0 male n = 3, female n = 2; day 4 male n = 2, female n = 3; day 6 male n = 3, female n = 2; day 8 male n = 9, female n = 7; day 10 male n = 3, female n = 2. (The data from these samples contributed to Fig. 6). Spearman correlation analysis showing R^2^ and *p* values. Error bars are standard error of the mean. Line is linear regression analysis with no designated intercept.

When male or female 4-month-old *IFNAR*^−/−^ mice were infected with the PRVABC59 strain of ZIKV, 16% of infected mice became paralyzed as measured by the viral paresis scale of 5 or 6^21^, 29% had observable behavioral motor deficits (viral paresis scale = 3 to 6), while 71% had no observable deficits (viral paresis scale ≤2) (Fig. 1A–D). The prevalence of motor deficits measured by viral paresis scale was between 27–33% (Fig. 1E,F) or 36–45% depending on the experiment (Supplementary Fig. S1). Motor deficits were also identified with the hanging wire test (Fig. 1G,H). All animals survived infection and recovered from motor deficits. Deficits had a rapid onset and recovery rate. Deficits peaked at 8–10 dpi (Fig. 1, Supplementary Fig. S1). The severity of deficits ranged from an obvious limp (viral paresis scale = 3) to full acute flaccid paralysis (viral paresis scale = 6). Viral paresis scale scores of 1 and 2 represent minor deficits that were sometimes scored for sham-infected animals by observers blinded to the identity of the mice. Thus, the limit of detection of ZIKV-induced deficits was a viral paresis scale score of >2.

Since only a subset of *IFNAR*^−/−^ mice develop motor deficits, as occurs in the human infection, we challenged mice with varying levels of virus to determine if the incidence was due to viral dosage or due to other factors. Escalating viral challenge dose (6.7 × 10^3^ pfu, 2.0 × 10^4^ pfu, or 6.7 × 10^4^ pfu per mouse) did not increase the severity or prevalence of hindlimb deficits (Supplementary Fig. S2). Diseased mice fully recovered usually within a week. A video of one mouse before development of paralysis (day 7), during paralysis (day 9), and during the recovery phase (day 16) (Supplement Video) is provided.

To determine if a more objective test could detect and confirm hindlimb deficits, we also employed the hanging wire test. This test was able to confirm the presence of deficits (Fig. 1G,H, Supplementary Fig. S2). There were some outlier values of sham-infected mice which may have been due to unrecognized behavioral factors other than motor deficits. The hanging wire test was correlated with the viral paresis scale assay where the sensitivity could be improved by increasing the maximum time from 60 seconds to 180 seconds (Supplementary Fig. S3). The correlation of the hanging wire test to viral paresis scale assay was significant (R^2^ = 0.76 and 0.26).

**Figure 2:**
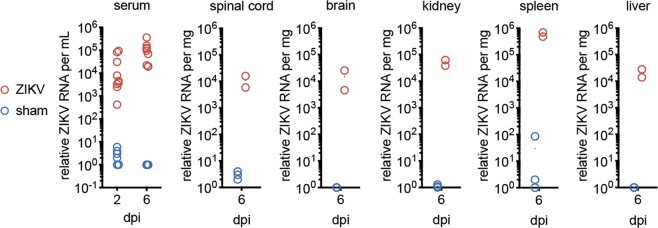
ZIKV RNA detected in serum, spinal cord and other tissues. RT-PCR specific for ZIKV was performed on serum collected from mice on 2- and 6-days post injection (dpi) (ZIKV-injected mice n = 6 males, n = 3 females and sham-injected mice n = 5 males, n = 2 females), and on tissues (brain, lumbar spinal cord, kidney, liver, spleen) from 2 ZIKV-infected mice (1 male, 1 female) collected on 6 dpi. As there were no sham tissues collected with this experiment, results were compared with tissues from sham-injected mice from a prior experiment.

To confirm that the virus is capable of productively infecting the older *IFNAR*^−/−^ mice, RT-PCR was performed on infected mice. ZIKV RNA was above sham-levels in the serum on 2 and 6 dpi. ZIKV RNA was also present in all tissues tested on 6 dpi (Fig. 2). Tracking viral load over time was not performed herein. These data, therefore, only demonstrated that the virus was present in the spinal cords of two paralyzed mice, but did not reveal the kinetics of viral load over time.

### Synaptic retraction in the spinal cord of paralyzed animals

**Figure 3:**
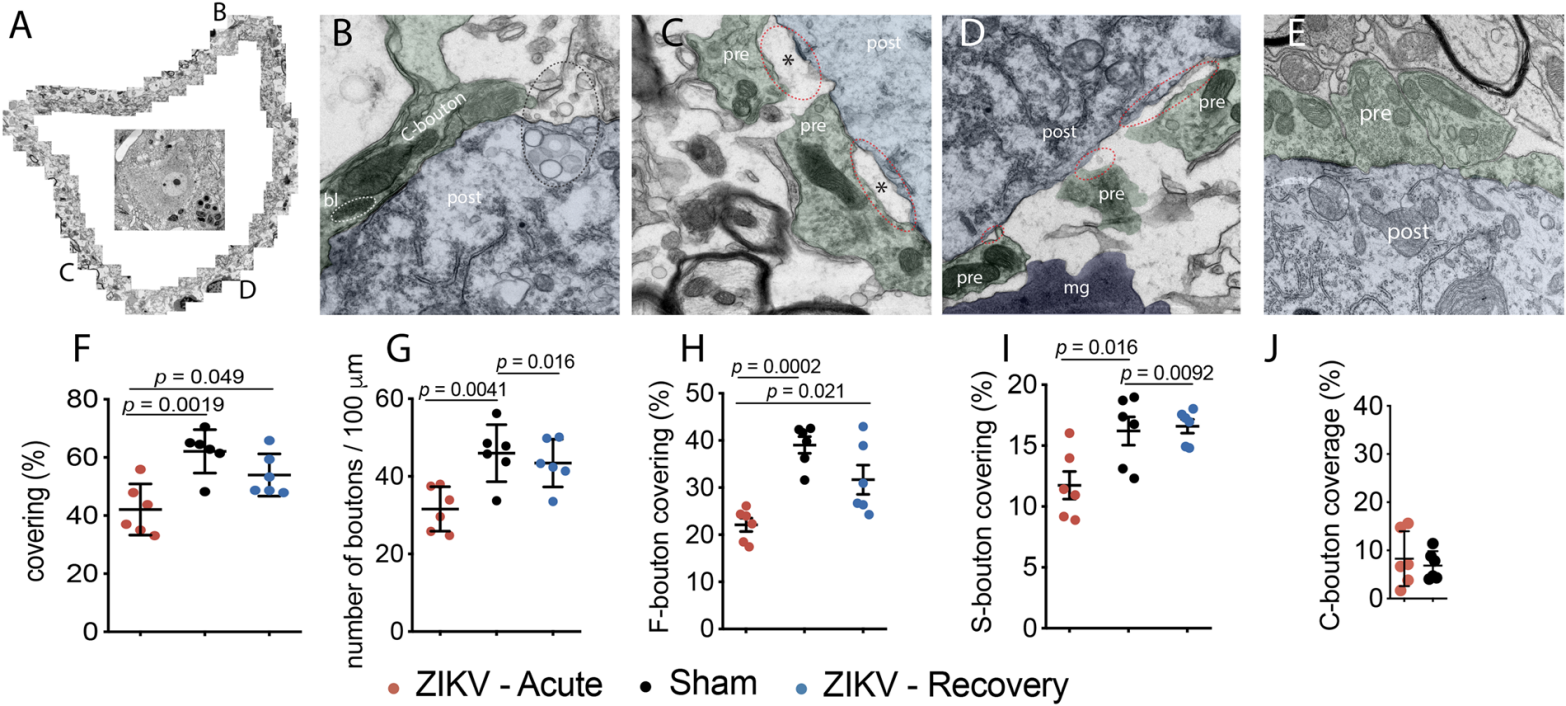
Synaptic retraction and morphological alterations of α-MN from male and female *IFNAR*^−/−^ mice, ages 3.2–3.9 months old. (**A**) Surface reconstruction of neuron in center from ZIKV-infected, paralyzed mouse. (**B**) Morphologically altered C bouton containing a group of blebbing vesicles (bl) near one mitochondrion. The bubbling (dashed ellipse) is associated with viral infected mice. (**C**) Retracted bouton (red ellipse circle) and putative astroglial projections (asterisks). (**D**) Retracted terminals (red ellipse circle) and microglia (mg, purple). (**E**) Sham-infected, (**F**) % covering of α-MN membrane, (**G**) number of boutons per 100 µm, (**H**) percent F-bouton (flattened-vesicle bouton with inhibitory inputs^20^) covering, (**I**) percent S-bouton (spherical-vesicle bouton with excitatory inputs^20^) covering, (**J**) percent covering C boutons (marker for α-MNs) of α-MN membrane. Seventeen MNs were from 6 ZIKV-infected paralyzed mice, 5 recovered mouse, and 6 sham-infected mice. pre: green pre-synaptic terminal, post: blue post-synaptic neuron. One-way analysis of variance was performed. 500-nm bar.

To determine if separation of pre-synaptic terminals from the post-synaptic motor neurons, or synaptic retraction, might be correlated with paralysis in this ZIKV temporary paralysis model, we counted the number of synapses around MNs in lumbar spinal cord-TEM images of ultra-thin sections. Some example images of synaptic retraction and altered morphology of MNs from a paralyzed animal (viral paresis scale = 6) (Fig. 3A–D) are shown compared to a sham-infected animal (Fig. 3E). Blebbing vesicles were present near mitochondria of motor-deficit mice (Fig. 3B). Quantification of 17 MNs (6 from paralyzed animals, 6 from a sham-infected animal, and 5 from recovered animals) indicated that percent covering of MNs with total boutons (Fig. 3F) and number of total boutons were reduced (Fig. 3G) in ZIKV-infected, paralyzed mice. To determine the types of boutons affected, the percent coverage of F, S, and C boutons were determined. F boutons were more strongly affected (*p* = 0.0002), and significantly started to rebound in recovered mice (*p* = 0.021) (Fig. 3H,J). S boutons were affected to a lesser extent (Fig. 3I,J), and C boutons, the marker used to identify αMNs, were not affected (Fig. 3J). The data suggested that synaptic retraction and re-association may be associated with paralysis and recovery, respectively. Astrocytic processes were seen interposed between retracted pre-synaptic terminals and the post-synaptic cell or perikarya (Fig. 3C), which may implicate astrocyte involvement in synaptic retraction.

### Dysfunctional motor neurons and possible proximal neuropathy detected by electrophysiology

**Figure 4:**
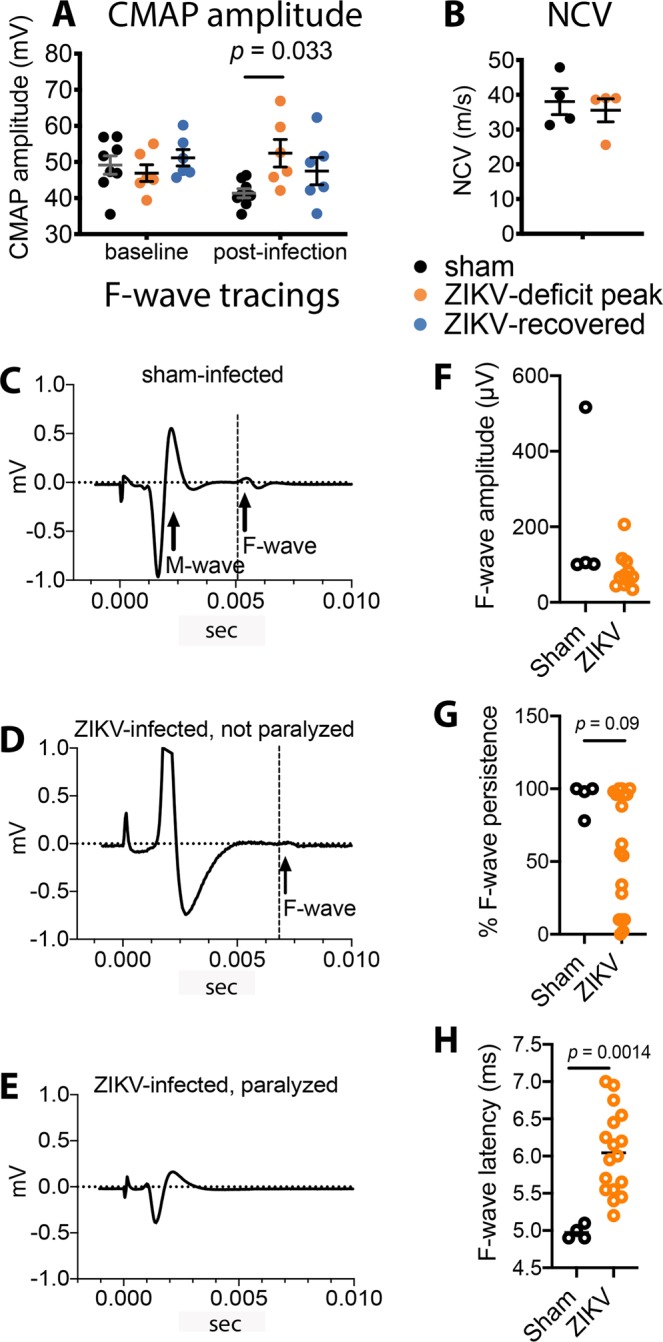
Compound muscle action potential (CMAP) and sciatic nerve conduction velocity (NCV) did not implicate distal neuropathy of the sciatic nerve, but F-wave latency may suggest proximal neuropathy. (**A**) Compound muscle action potential was not decreased but was significantly increased during ZIKV-induced motor deficits. (**B**) Nerve conduction velocity was not affected during motor deficits. F-wave tracings from (**C**) sham-infected mouse (**D**) ZIKV-infected, not paralyzed mouse, and (**E**) ZIKV-infected, paralyzed mouse. (**F**) F-wave amplitude, (**G**) % F-wave persistence, and (**H**) F-wave latency. Two males at 7.3-months-old, two males and two females at 5.9-months-old and 3 females at 3.2-months-old were injected with ZIKV and one each male and female at 7.2-month-old were injected with cell culture lysates for controls. At days 9 and 10 after viral challenge, % F-wave persistence and F-wave latencies were measured. Data points are of individual hindlimbs. (**A**) One-way analysis of variance was performed. (**F**,**G**) T-test was performed.

To help localize the lesions responsible for motor deficits, we performed electromyography on the left and right hindlimbs. Compound muscle action potentials of ZIKV-infected animals were not decreased compared to that of shams (Fig. 4A), rather they were increased in paralyzed animals. As a measure of how fast electrochemical impulse moves through the nerve and as a diagnostic marker for neuropathy, nerve conduction velocities were determined. Nerve conduction velocities, where stimulation was induced in the sciatic notch or ankle, were not affected in paralyzed mice compared to sham mice (Fig. 4B). Data from both these assays suggest normal nerve function.

F-waves could readily be detected in hindlimbs of sham-infected mice (Fig. 4C); but they were absent in 3 of 4 hindlimbs of paralyzed mice (viral paresis scale = 6) even with the presence of M-waves (Fig. 4E). F-waves could be detected in ZIKV-infected mice without overt paralysis (Fig. 4D). Since we observed that F-waves were not detected in some paralyzed mice (Fig. 4E), we calculated amplitudes from two different experiments (Fig. 4F, Supplementary Fig. S8). The differences in amplitudes between the ZIKV- and sham-infected mice using the T-test were not statistically different, which was possibly due to high amplitude-variability. Nevertheless, many of the amplitudes from ZIKV-infected mice were lower than the sham-infected mice. Only 3/12 (25%) (Fig. 4G) of the values from ZIKV-infected mice in one experiment and 2/7 (28%) (Supplementary Fig. S8) in the other experiment were at the levels of sham-infected mice. Therefore, most of the ZIKV-infected mice had amplitudes lower than the lowest amplitudes of control mice, which may have reflected motor neuron dysfunction.

Percentage F-wave persistence was diminished in ZIKV-infected mice compared to controls (Fig. 4G), but not to a statistically significant level. Due to the low numbers of sham-infected mice, we conducted a second experiment wherein the % F-wave persistence of ZIKV-infected, motor impaired mice was statistically lower (*p* = 0.007) than data of control mice (Supplementary Fig. S8). The increased % F-wave persistence in motor-impaired mice may have also reflected dysfunctional MNs in ZIKV-infected mice.

The F-wave latencies of hindlimb nerves from ZIKV-infected mice were statistically increased compared to nerves of sham-infected mice in two separate experiments (*p* = 0.0014, Fig. 4H), *(p* = 0.015, Supplementary Fig. S8). Since the distal nerve from the sciatic notch to muscle appeared normal by nerve conduction velocity (Fig. 4B), the possible neuropathy contributing to the increased F-wave latency was possibly located near the spinal cord proximal to the sciatic notch.

## Histology

### Spinal MNs do not die

**Figure 5:**
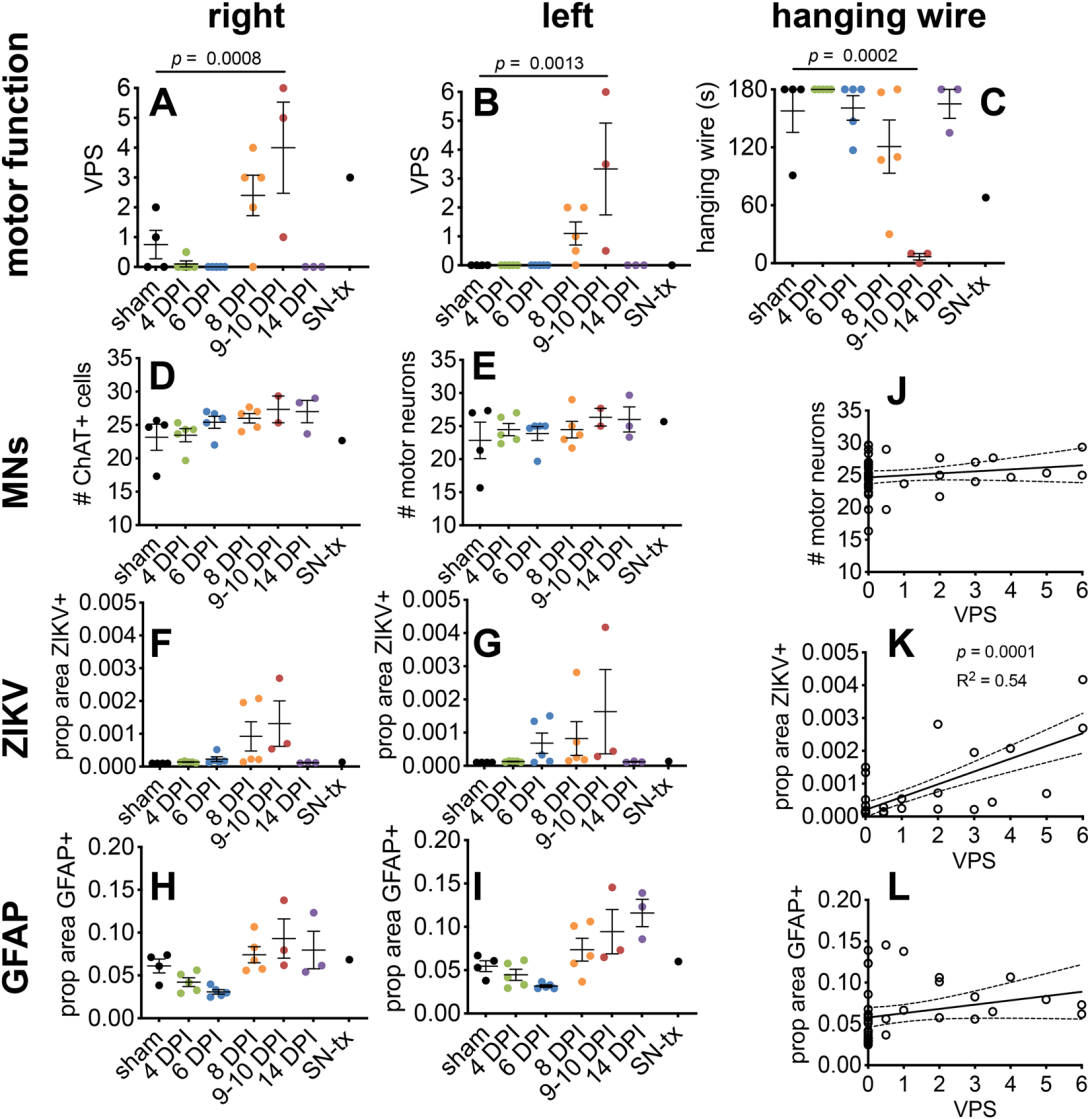
Correlation of motor deficits with ZIKV immunoreactivity in the spinal cord with no loss of motor neurons. (**A,B**) viral paresis scale (VPS) assay, (**C**) hanging wire assay. Quantification of (**D,E**) motor neurons, (**F,G**) ZIKV, (**H,I**) astrocytes. (**J,K,L**) Correlation with viral paresis scale. Statistical analysis (**A**–**C**) one-way analysis of variance with Dunnett’s multiple comparisons test, and (**J**–**L**) best-fit linear regression analysis using GraphPad software. Male (n = 17) and female (n = 16) *IFNAR*^−/−^ mice between the ages of 5.0- to 5.3-months-old were injected with 6.7 × 10^3^ pfu of ZIKV. Five animals (black, n = 3 male, 2 female) received a sham injection of cell homogenate. Two uninfected animals (females) received a sciatic nerve transection on the right hindlimb. Prop area refers to proportional area. Right refers to right hindlimbs (**A**) and to right hemi-sectioned spinal cords (**D,F,H**). Left refers to left hindlimbs (**B**) and to left hemi-sectioned spinal cords (**E,G,I**). Animals were perfused with freshly made paraformaldehyde at 4 dpi (green, n = 2 males, 3 females); 6 dpi (blue, n = 3 males, 2 females); 8 dpi (orange, n = 3 males, 2 females); 9–10 dpi, the day of peak VPS deficit, (red, n = 2 males, 1 female); and 14 dpi (purple, n = 1 male, 2 females with recovered VPS scores after having deficits on the right/left hindlimbs of 4/1, 1.5/3, 3/0.5). Cords from sham-animals were also collected (n = 3 male, 1 female). One sciatic nerve-transected animal (black, Sn-Tx) was perfused at 7-days after transection for a control comparison. Each data point in (**D,E,F,G,H,I**) is the average of two sections from a single mouse^66^. One-way analysis of variance was performed.

Since neurotropic flaviviruses notoriously infect and destroy motor neurons^22–24^, we determine if death of MNs by ZIKV was also responsible for paralysis. ZIKV-infected *IFNAR*^−/−^ mice were necropsied at various timepoints to analyze cross-sections of the lumbar spinal cord stained with antibodies against ZIKV, ChAT to label MNs, and GFAP to label astrocytes. Motor deficits occurred between 8–10 days in 37% of ZIKV-infected mice after viral challenge and all mice recovered within the following week (Fig. 5A–C). Even during times of motor deficits or paralysis, ChAT immunoreactive MNs did not decrease in numbers, indicating that they did not die. This was verified in two different experiments (Fig. 5D,E, Supplementary Fig. S4).

ZIKV immunoreactivity was first apparent in lumbosacral spinal cord sections at 6 dpi, peaked at 9–10 dpi, and was gone by 14 dpi (Fig. 5F,G). To determine if ZIKV, astrocytes, or loss of MNs were most closely associated with motor function loss, correlation analysis was performed for ZIKV, GFAP, or ChAT immunoreactivities vs viral paresis scale scores. Of the three immune-reactive markers, ZIKV immunoreactivity were most correlated (*p* = 0.0001, R^2^ = 0.54) with hindlimb motor deficits (viral paresis scale scores) (Fig. 5J–L).

**Figure 6:**
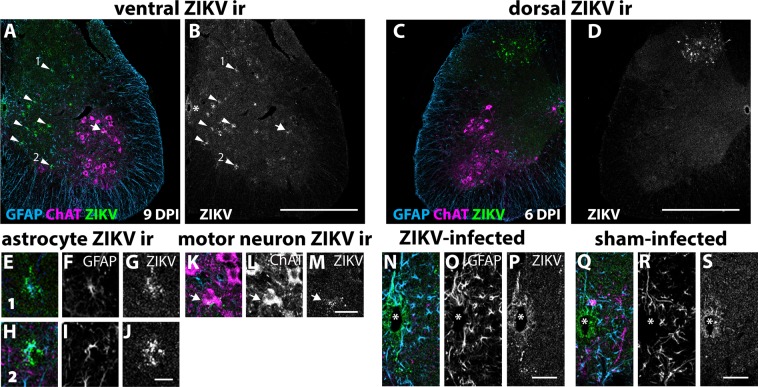
ZIKV immunoreactivity (ir) was mostly spotty and diffuse in the (**A,B**) ventral horn, occasionally in the (**C,D**) dorsal horn, in many **(E-G, H-J)** astrocytes (arrow head) and (**K**–**M**) rarely in motor neurons (arrow). #1 and #2 arrow heads in (**B**) correspond to (**E**–**G, H**–**J**) respectively*.* Single arrow in (**B**) corresponds to (**K**–**M**) of ZIKV ir co-localized with ChAT immunoreactivity. ZIKV ir was present around the central canal (asterisk) in ZIKV- (**N**–**P**) and sham-infected mice (**Q**–**S**), so the staining was non-specific around the central canal. *IFNAR*^−/−^ mice, ages 5.0–5.3 months old, were injected with 6.7 × 10^3^ pfu of ZIKV. Sham-infected mice were injected with cell culture homogenates. Bars 50 nm.

ZIKV immunoreactivity was mostly spotty and diffuse in the lumbosacral spinal cord of paralyzed mice. The ZIKV immunoreactivity was present in the ventral horn (Fig. 6A,B) and occasionally in the dorsal horn (Fig. 6C,D). The identification of cells co-localized with ZIKV immunoreactivity was often unclear, but some staining was associated with astrocytes (Fig. 6E–J). Only rarely was the ZIKV immunoreactivity associated with ChAT + neurons (K-M). The ZIKV immunoreactivity was subjectively much lower than previously observed in younger AG129 mice infected with ZIKV where the disease was lethal^21^. To definitely measure the viral load and tropism, more comprehensive future studies would be required.

### Inflammation and astrogliosis

Analysis of GFAP labeling in two different experiments demonstrated that astrogliosis was present in paralyzed animals but did not diminish in recovered animals (Figs. 5H,I, and S4). Astrocytes also appeared to be possibly infected by ZIKV (Fig. 6D–I, arrowheads).

Inflammatory cells were assessed with antibodies against iba1 to label microglia/macrophages, CD3 to label T-cells, and Ly6G to label neutrophils. Trends of increasing Iba1 immunoreactivity were identified in two different experiments, but increasing levels of CD3 immunoreactivity was not consistent between two experiments (Figs. S5 and S7). The amoeboid morphology of iba1 + cells in the spinal cord of paralyzed mice was suggestive of infiltrated macrophages or fully activated microglia (Supplementary Fig. S5). Ly6G immunoreactivity (neutrophils) was not detected in two sets of experiments (Supplementary Figs. S5 and S7). As a positive control, Ly6G antibody did label neutrophils in positive-control spleen tissue (Supplementary Fig. S5).

As a control, the sciatic nerve was transected and then sectioned for analysis 7 days later. This showed the expected patterns: hindlimb deficits on the side of the transected hindlimb, decreased hanging wire time (Fig. 5C, labeled as SN-tx), no ZIKV (Fig. 5F), astrogliosis (Fig. 5H), and possible reduction of the number of MNs on the side of transection compared to other groups of mice (Fig. 5D).

### Levels of the pre-synaptic marker, synaptophysin, were not decreasd, but were increased in the spinal cords of paralyzed animals

**Figure 7:**
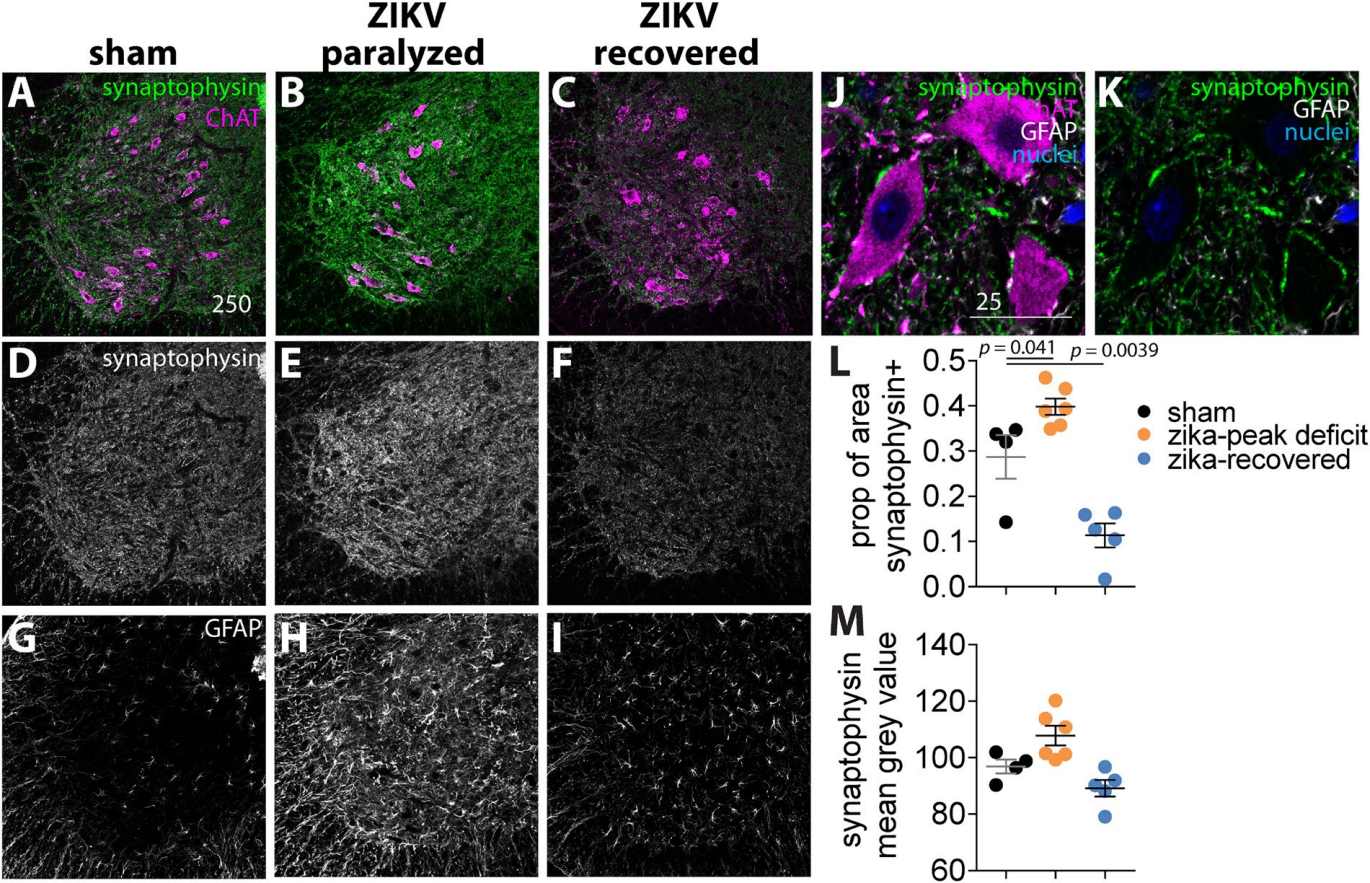
Levels of the pre-synaptic marker, synaptophysin, were not decreased, but were increased, in the spinal cords of paralyzed animals. IHC immunoreactivity (ir) of (**A,D,G**) sham-infected mouse, (**B,E,H**) ZIKV-infected, paralyzed mouse, (**C,F,I**) ZIKV-infected, recovered mouse. (**A,B,C**) merged images of synaptophysin ir and ChAT ir, (**D,E,F**) synaptophysin ir, (**G,H,I**) GFAP ir. Close up of (**J**) synaptophysin ir, ChAT ir, GFAP ir, and DAPI, (**K**) synaptophysin ir, GFAP ir, and DAPI. Quantification, (**L,M**) quantification of synaptophysin ir. All *IFNAR*^−/−^ mice were males (4.0–4.2 months old). Sham n = 4, ZIKV paralyzed n = 5, ZIKV recovered n = 5. SN-tx: transected sciatic nerve control. Each data point in (**L,M)** is the average of two sections per mouse^66^. One-way analysis of variance was performed. Bars = 250 µm or 25 µm.

To determine if rapid changes to the amounts of synapses around MNs is associated with paralysis, lumbar spinal cord sections were stained with synaptophysin, a pre-synaptic marker. Synaptophysin staining was diminished during the paralysis phase in EAE rat models^20^. Interestingly, the proportion of synaptophysin-positive pixels was significantly increased in paralyzed animals (*p* = 0.041) and decreased in recovered animals (*p* = 0.0039) compared to sham-infected controls (Fig. 7). This suggests that the number of synapses around MNs was not reduced during ZIKV-induced temporary paralysis as in the EAE model.

### Sciatic nerve was not infected, and axons and myelin sheaths did not appear to be structurally altered in paralyzed animals, although there was some evidence of an inflammatory response

Analysis of cross sections of the sciatic nerve corroborate electrophysiological findings for a lack of distal peripheral neuropathy. The morphology of axons and myelin as revealed by neurofilament (NF) and myelin basic protein (MBP) labeling appeared normal in paralyzed animals (Supplementary Fig. S6). Additionally, there was no detectable ZIKV by IHC (Supplementary Fig. S6), at least within the limits of detection. The cross-sectional area of the nerve was normal (Supplementary Fig. S7). While there was a trend of macrophage infiltration in paralyzed animals, as revealed by iba1 labeling, the level did not reach statistical significance (Supplementary Fig. S7). There was no evidence of T cell (CD3) or neutrophil (Ly6G) infiltration in the nerve. As a positive control, the antibodies labeled cells in a positive control spleen tissue (Supplementary Fig. S7).

Human Schwann cells play a central role in peripheral nerve functions and have been identified to be susceptible to ZIKV infection in culture. Their biological responses, including cytokine responses, are altered^25^, but the relationship to development of human Guillain-Barré syndrome is unknown. Within the limits of detection, we did not identify obvious ZIKV immunoreactivity in cells associated with peripheral sciatic nerves of these mice. If infection of human Schwann cells occurs in human patients to contribute to the development of Guillain-Barré syndrome, other co-factors such as synaptic retraction may be related to the development of temporary paralysis.

### Gastrocnemius muscle was not ZIKV-infected, and there was no neutrophil response related to viral infection

H&E stained sections, analyzed by a board-certified pathologist, indicated neutrophil infiltration was present in both ZIKV- and sham-infected mice along needle tracts likely due to the injury caused by the EMG needles used for electrophysiology. Infiltration was not observed elsewhere. Analysis of muscle sections with antibodies against ZIKV did not reveal ZIKV labeling in muscle tissue of infected or sham animals.

## Discussion

We showed that ZIKV can cause a temporary acute flaccid paralysis in mice that recover within a week, and that paralysis was associated with infection of the spinal cord without the destruction of MNs. To directly assess the pathological effects on α-MNs, the ultrastructure of α-MNs was evaluated in infected paralyzed, and recovering mice, and in sham-infected mice. α-MNs from paralyzed mice had a statistically significant increase in the number of boutons separated from the MNs, a process we referred in the report as synaptic retraction, or referred to in other reports as synaptic plasticity, synaptic detachment, or synaptic rearrangement^19,26–29^. The correlation of synaptic retraction with paralysis and its subsequent synaptic normalization with recovery from paralysis was particularly significant, because detached synapses from the cell bodies would cause impaired function of MNs and re-association of synapses will help restore function of MNs during recovery of paralysis.

Studies with West Nile virus (WNV), simian immunodeficiency virus (SIV), human immunodeficiency virus (HIV), and a human endogenous retrovirus (HERV-K) provide evidence of viral induced synaptic rearrangement. Post-mortem human and murine samples of the hippocampal CA3 had a loss of presynaptic terminals^30^. This was associated with memory impairment in mice. Astrocytes in encephalitic SIV-infected animals had decreased arbor length in the white matter and reduced complexity in grey matter, which may lead to changes in synaptic structure and function^31^. Using synaptophysin as a marker for synaptic structure with confocal microscopy, neurite retraction was identified and was determined to be mediated by HIV-1 Tat protein through inhibition of cellular factors^32^. Also, neurotoxicity studies of the cerebral cortex and spinal cord in transgenic mice expressing the *env* gene of HERV-K revealed retraction and beading of neurites. Data of open-field electrophysiological testing also provided supporting data of neurite retraction. The expression of the HERV-K or its protein in ALS patients suggested that these phenotypic changes may contribute to neurodegeneration. In this report, we show the first direct electron microscopic evidence of synaptic retraction of spinal motor neurons *in vivo* caused by a viral infection.

Because the human assays used to monitor Guillain-Barré syndrome are far more detailed and standardized compared to the assays used with small-sized mice, comparisons between this mouse model and human Guillain-Barré syndrome are incomplete. For example, standard-of-care procedures and normative electrophysiological values are available for human patients to know if values are abnormal from the population. Nevertheless, some broad, useful comparisons can be made between human Guillain-Barré syndrome and the ZIKV-induced temporary paralysis of this mouse model (Table 1). Acute flaccid paralysis and increased F-wave latencies occur in both diseases. The acute flaccid paralysis in ZIKV-associated Guillain-Barré syndrome is bilateral. The acute flaccid paralysis in the mouse model may also be bilateral, since mice with overt paralysis generally had some motor impairment on the other limb. Both diseases involve recovery from paralysis, which occurs in both the mice and human. Recovery of paralysis in the mouse within a week is remarkable. The timeline for recovery from paralysis in the mouse model is shorter compared to weeks or months with human Guillain-Barré syndrome^33^.

**Table 1:** Comparison of ZIKV-associated temporary flaccid paralysis with Guillain-Barré syndrome (GBS)^33,65^.

Disease phenotypes	ZIKV-associated paralysis of mice	Human GBS, AIDP^a^
bilateral, flaccid paralysis	+/−	+
recovers from paralysis	+	+
distal peripheral neuropathy	−	+
nerve conduction velocity	−	+
inflammatory neuropathy	increased	+/−
F-wave latency	increased	increased
synaptic retraction	+	nd
myelitis	mild	undetected
MN death	−	nd
MN infection	nd	nd
deep tendon reflexes	nd	decrease
monophasic time course	+	+
CSF high protein, low cell #	nd	+

^a^AIDP – acute inflammatory demyelinating polyneuropathy.

Peripheral neuropathy is a hallmark criterion for diagnosing human Guillain-Barré syndrome. More specifically, if nerve conduction velocity is less than 90% of lower normative values, it can be added to one of three criteria to diagnose peripheral demyelinating neuropathy^33^. This temporary paralysis mouse model does not meet this criterion, since there is no reduction of sciatic nerve conduction velocity. If F-wave latency exceeds 125% of upper limit of normative human values, it can be added as a second criterion for diagnosing demyelinating neuropathy. The mouse model of this study reflects this criterion by having increased F-wave latencies.

The earliest electrophysiological abnormalities detected in the most common type of Guillain-Barré syndrome associated with ZIKV outbreaks (acute inflammatory demyelinating polyneuropathy, AIDP) are increased F-wave latencies or poor repeatability of F-waves (F-wave persistence)^34^. This is because nerve roots are affected earlier in the development of AIDP than distal peripheral nerves. Since the nerve conduction velocity assay used in this study measured nerve function distal to the sciatic notch or ankle, one could not determine the status of neuropathy near the spinal cord. However, the F-wave latency relies on the health of the entire nerve including the nerve roots and nerve rootlets, since they are in the electrochemical circuit of F-waves. Consequently, the possible neuropathy identified by increased F-wave latency was possibly located proximal to the sciatic notch near nerve roots or rootlets.

A combination of peripheral neuropathy and MN synaptic retraction may contribute to paralysis. Microscopically, mild myelitis was seen in the spinal cords of the ZIKV-induced paralysis mouse model. It is possible, therefore, that the mild myelitis associated with synaptic retraction may not be sufficient to be detected by imaging in human patients.

Further studies will be required to determine if ZIKV-induced synaptic retraction observed in this temporary paralysis model actually causes or contributes to the paralysis phenotype and if transient synaptic retraction in combination with peripheral neuropathy is involved with human ZIKV-induced temporary paralysis. To our knowledge, no studies have determined the extent of synaptic retraction or detachment required to induce flaccid paralysis, so we do not know if the levels of retraction observed herein could have a paralytic phenotypic effect.

EAE relapsing paralysis models of multiple sclerosis (MS) in mice and rats manifest synaptic retraction from MNs that is coincident with the onset of paralysis, but abates during recovery, whereas, demyelination changes are not coincident with paralysis^19,20,29,35^. Investigators^19^ advocate that these rapid synaptic changes could account for temporary quadriplegia in MS. In the EAE models, recovery rapidly occurs within a week of paralysis, which is similar to remission of quadriplegia in MS patients and with the viral paralysis mouse model. Since TEM is considered the gold standard for evaluating synaptic changes, we evaluated α-MNs and their synapses in an effort to correlate clinical motor deficits of ZIKV infection in mice and ultrastructural alterations at the MN of the spinal cord.

Typing the excitatory and inhibitory boutons is important, because in the temporary paralysis EAE model, the ratio of excitatory-to-inhibitory boutons changes to favor of inhibitory boutons that could suppress motor function^20,28^. Also, α-MN can be identified by the presence of C-type boutons in TEM^36^. Of note, the most affected terminals following peak dysfunction are inhibitory, namely F-boutons. This is in line with previous observations following traumatic lesions, such as sciatic nerve crush^28^, and spinal ventral root avulsion^37,38^, which may in turn affect motor coordination. Glutamatergic terminals fully recover, suggesting that following paralysis, proprioceptive inputs are restored spontaneously.

### Synaptic retraction may be a neuroprotective response

Some have hypothesized that synaptic retraction may be a normal and protective response to various spinal cord injuries^20^, and inflammatory reactions during EAE^19^, which may also be applicable to ZIKV infection of the spinal cord. During EAE inflammation, the synaptic transmission to MNs becomes temporarily impaired, possibly causing paralysis. Soon after paralysis, synapses return to apposition and motor functions are quickly restored^19^. Spinal cord injury can elicit glutamate excitotoxicity, which is due to excess levels of the excitatory neurotransmitter glutamate, that allows lethal levels of Ca^2+^ to enter neurons^20^. Spatial separation of pre-synaptic terminals from neurons may suppress activation of ion channels and the deleterious effects of high intracellular calcium. Conversely, without synaptic retraction, toxicity of the pathogenic processes may destroy MNs to cause permanent paralysis.

Flaccid paralysis is usually caused by damage to peripheral nerves or to MN function of the spinal cord, whereas, spastic paralysis involves CNS lesions in the brain or spinal cord; therefore, the flaccid paralysis of this mouse model is more likely caused by spinal cord lesions. In support of this, preliminary analysis of sections of motor cortex, cerebellum and the brainstem revealed very little immunopathogenesis with only rare ZIKV immunoreactivity and astrogliosis, some macrophage/microglia infiltration, and very few T cell or neutrophil infiltration. Additionally, F-waves were not observed in 3 of 4 limbs assayed from ZIKV-paralyzed mice. Since F-waves cannot be detected if MNs are severely dysfunctional, the lack of F-waves in paralyzed mice provides additional evidence that spinal cord lesions contribute to paralysis.

*IFNAR*^−/−^ mice were used in these studies, because adult wild-type laboratory mice are not susceptible to ZIKV infection. A rapid series of publications following the ZIKV outbreak found that adult mice that lack type 1 (IFN α/β; A129 and *IFNAR*^−/−^ strains) or types 1 and 2 (α/β/γ; AG129 strain) interferon receptors are susceptible to lethal infection^39–43^. These models may have flaws, but they may have some relevance to human ZIKV infections in that, like many viruses, ZIKV gains advantages in human hosts by inhibiting interferon responses^44–46^. Because the virus may not be able to inhibit mouse-specific interferon pathways^47^, blocking them by other means may more closely mimic what happens in humans. Although *IFNAR*^−/−^ mice are deficient in type 1 interferon responses, they do elicit type 2 interferon and acquired immune responses as noted in vaccine-elicited protective immunity^48,49^. In this study, ZIKV infection of 4-month-old *IFNAR*^−/−^ mice was not lethal as compared to infection of young *IFNAR*^−/−^ mice.

In summary, the transient paralysis observed in these ZIKV-infected mice was associated with non-cytolytic α-MN pathologies including spatial separation of pre-synaptic nerve terminals from the post-synaptic α-MN, which begin to normalize upon recovery. Electrophysiological evidence also suggests peripheral neuropathy near the spinal cord may occur, which could also contribute to paralysis. Reversible synaptic retraction may be a previously unrecognized cofactor with peripheral neuropathy for the development of acute flaccid paralysis in Guillain-Barré syndrome associated with ZIKV infection.

## Methods

### Animal welfare

This study was conducted in accordance with the approval of the Institutional Animal Care and Use Committee of Utah State University. The work was done in the AAALAC-accredited (reference file #000649) Laboratory Animal Research Center of Utah State University. The U.S. Government (National Institutes of Health) approval was renewed (Assurance no. A3801–0) in accordance with the National Institutes of Health Guide for the Care and Use of Laboratory Animals (Revision; 2010).

### Animals

Adult male and female interferon αβ-receptor knockout (*IFNAR*^−/−^) mice (Jax stock # 010830)^50^ were bred in-house in sterilized isolator cages maintained in a 12/12 light cycle. Mice were randomly assigned to treatment groups based on weight, gender, and baseline measurements.

### Virus

A Puerto Rican isolate of ZIKV (PRVABC59, Human/2015/Puerto Rico, GenBank: KU501215) is described^21^. One-half log serial dilutions (6.7 × 10^4^ pfu, 2 × 10^4^ pfu, 6.7 × 10^3^ pfu, 2 × 10^3^ pfu per 100 µL) were made in minimal essential medium supplemented with 50 μg/mL gentamicin for subcutaneous injection of 100 μl in the inguinal area on the right side of the mice. Uninfected cell lysates were prepared and diluted similarly for sham injections. The ZIKV RT-PCR is described^51^. The limit of detection was calculated based on the titer values from sham or uninfected testis tissue.

### Behavioral motor function

Mice were analyzed for signs of hindlimb paresis/paralysis using the viral paresis scale^21^. The scores are 0: normal: normal, weight-bearing, plantar stepping; 1: onset of symptoms: mild miss-step (rotation); 2: mild paresis: mild miss-step (toe-curling, slight skidding), slight limp; 3: moderate paresis: obvious miss-step (foot curling, obvious skidding medially or laterally), obvious limp; 4: severe paresis: limb mostly dragging, not much weight bearing, still helps with forward motion; 5: paralysis: limb dragging, no weight bearing, slight joint movement; 6: complete paralysis: limb dragging, no weight bearing, no joint movement.

The hanging wire test was performed as described^52,53^. Mice were placed on a wire netting, which was inverted and held 50 cm above a fresh cage containing soft bedding. The length of time the hindlimbs remained on the wire mesh was recorded (in seconds) up to 60 or 180 seconds (s), depending on the experiment. If animals hung on for the maximum time, 60 or 180 s, the animals were returned to their home cage. If the hindlimbs fell before the maximum time, the animals were tested for 2 more trials (3 total) with a 2-min rest between trials. The average time across the 3 trials was then recorded.

### Electromyography

The compound muscle action potential was performed as described^21^, except that monopolar needle electrodes (Protectrode, # PRO-37SAF, The Electrode Store, Enumclaw, WA) were used to stimulate the sciatic notch. Muscle responses were recorded with custom-made ring electrodes with contacts coated with electrode gel. The animal ground was another custom-made ring electrode connected to the animal’s tail. Data were acquired at a 40 K/second sampling rate with Powerlab 4/25 and LabChart 8 software (ADInstruments).

For nerve conduction velocity, compound muscle action potential was obtained for the gastrocnemius and flexor digitorum brevis muscles. The muscles were stimulated at the sciatic notch (distal site) and then at the tibial nerve on the medial side of the ankle (proximal site) with 0.1 or 0.2 ms pulses of current. The voltage was increased incrementally until a maximum amplitude was reached. Determination of nerve conduction velocity was calculated as described^54^.

The % F-wave persistence was developed using published F-wave tracings^55–57^. The stimulating cathode and anode monopolar electrodes (EL452, Biopac Systems, Inc., Coleta, CA) were inserted and stabilized at the tibial nerve of the ankle. Recording electrodes (sterile acupuncture needles, size 0.25 × 13, Tai Chi Brand, distributed by Lhasa OMS, Inc., Weymouth, MA)^58^ were inserted between the digits of the 2^nd^ interosseous muscle and the reference acupuncture electrode was inserted between the digits of the 3^rd^ interosseous muscle. The ground electrode was placed at the base of the tail^57^. Repeated stimulations of 5 volts (V), 0.2 Hz frequency were used^55^. The percentage of persistence of F-waves was calculated from the number of F-waves detected with 50 stimulations.

For data in Fig. 4, the F-wave latency was obtained under super-maximal stimulation (~2.5 times of F-wave threshold) from measuring the time (ms) between the stimulation artifact to the onset of F-wave tracing. An average of 5 repeated measurements were reported. For data in Supplementary Fig S8, the stimulation voltage was increased incrementally until the maximal F-wave amplitudes were achieved. The average F-wave latencies of 5 stimulations was reported. The amplitudes were measured from peak-to-peak using the F-wave tracings.

### Histological analyses

We analyzed sections containing motor neurons supplying innervation in the hindlimbs of mice from rostral L4 section to caudal of L5^59^ using a mouse spinal cord atlas^60^. The L5 was identified at the T13 vertebrate of the spine and by tracing the 13^th^ rib^61^. Two 25-µm sections separated by 10 sections were analyzed. Quantification of each hemisphere was averaged for each mouse. Each data point represented a single mouse. Mouse perfusion, cryopreservation, immunofluorescence staining, confocal microscopy and quantification by ImageJ™ (version 1.51J8, National Institutes of Health, USA) are described^21^. Primary antibodies were diluted in blocking solution (see Table 2) and applied to sections for incubation overnight at room temperature. For CD3 and Ly6G labeling, the amount of Triton X-100 was decreased to 0.5% in the blocking solution and 0.2% in the antibody diluent. For synaptophysin labeling, the blocking solution was PBS with 20% normal serum, and no Triton X-100, and the antibody diluent was PBS with 10% serum and 0.3% Triton X-100. Sections were incubated with primary antibody over two nights at 4 °C.

**Table 2:** Summary of primary antibodies and dilutions used.

Antibody	Antibody Type	Company, Catalogue #	Dilution
ChAT	goat pAb	Millipore, AB144P	1/100
ZIKV	rabbit pAb	IBT, 0308-001	1/500
iba1	goat pAb	Abcam, ab5076	1/200
GFAP	rat IgG2a-kappa mAb	Invitrogen, 13-0300	1/500
CD3	rabbit mAb	Abcam, ab16669	1/100
NF-H	chick pAb	Aves Labs, NFH	1/200
MBP	rat IgG2a mAb	Abcam, ab7349	1/200
Ly6G	rat IgG2b mAb	Abcam, ab25377	1/500
Synaptophysin	rabbit MAb IgG	Fisher, PIMA516402	1/100

Abbreviations: pAb, polyclonal antibody; mAb, monoclonal antibody.

For tyramide amplification of ZIKV, endogenous peroxidases were quenched with 0.3% H_2_O_2_ in PBS for 30 min and rinsed twice with PBS before the blocking step. After labeling with a secondary antibody conjugated to horse-radish peroxidase, sections were rinsed with PBS containing 0.05% triton (PBST), then incubated with a 1/50 dilution of tyramide (Invitrogen, T20949, Alexa568 conjugated, Invitrogen, Carlsbad, CA) diluted in tyramide diluent (0.1 M borate buffer, pH 8.5 containing 0.003% H_2_O_2_) for 10 min. Sections were then rinsed 5 times with PBST for 5 min each before proceeding to Hoechst staining.

### Imaging and image processing

Eight-bit fluorescent images were obtained at room temperature with a laser scanning confocal microscope (Zeiss, LSM710, Thornwood, NY) equipped with 405, 488, 561, and 633 laser lines and Zen image acquisition software. Objectives used were 10 × (NA 0.45), 20 × (NA 0.8), 40X oil (NA 1.4). For images taken for pixel-based quantification, identical settings were used for all images in a set. For images chosen for publication, distracting artifacts were removed in ImageJ^62^ and levels were adjusted in Adobe Photoshop to maximize the signal-to-noise ratio so that relevant features could be seen more clearly. For images chosen to highlight pixel-based quantification, sham- and ZIKV-group images were adjusted identically to enable equitable comparison.

### Image quantification

ImageJ was used for image quantification^62^. The cell counter plugin was used for manual cell counts. For pixel-based quantification of a given antibody signal, the area to be measured was defined by a region of interest (ROI), after which thresholds were established in single channel images to select pixels with a positive signal (positive pixels). The area of positive pixels was divided by the whole area of the main ROI to determine what proportion of the ROI was positive for the antibody.

Spinal cord levels were identified using the mouse spinal cord atlas^63^, and the location and morphology of clusters of choline acetyltransferase (ChAT)-positive neurons. Because MNs for the gastrocnemius muscle are located at the L4-L5 level^59^, sections from this level were chosen for analysis when possible.

In one experiment, two sections per spinal cord, spinal cord hemisection or sciatic nerve were quantified and averaged. The left and right side of the spinal cord was distinguished only for synaptophysin quantification, whereas, both sides were quantified for all other spinal cord analyses. Thus, the MN number was the total number of MNs in the section. Right and left sciatic nerves were distinguished and quantified separately. In a second experiment, three sections per spinal cord hemisection were quantified and averaged. More care was taken during sectioning to ensure that the right and left sides of the spinal cord could be distinguished.

### TEM analysis

Mice were perfused transcardially with phosphate buffered saline (PBS) followed by freshly made, room temperature EM fixative (1% paraformaldehyde, 2.5% glutaraldehyde, 3% sucrose w/v, 0.0012% CaCl2, 0.1 M phosphate buffer (PB), pH 7.4). The lumbar spinal column was removed and placed in cold EM fixative and post-fixed overnight rocking at 4 °C. After a few rinses in 0.1 M PB, a 1-mm thick cross-section of the L4-L5 spinal cord was dissected from the spinal column and post-fixed in EM fixative at 4 °C so that the total post-fix time was at least 48 hours. Samples were transported to the University of Utah EM core for further processing. Samples were rinsed in 0.1 M PB, post-fixed with 2% osmium tetroxide for 2 hours at room temperature, rinsed in nanopure water, stained with saturated aqueous uranyl acetate for 1 hour at room temperature, dehydrated through a graded ethanol series, and infiltrated with plastic before embedding in EMbed 812 resin (Electron Microscopy Sciences, Hatfield, PA) for cross-section. The left and right sides of the spinal cord were embedded in separate blocks and transported to the A. L. R. Oliveira lab in Universidade Estadual de Campinas (UNICAMP), Brazil.

Neurons with large cell bodies (>35 μm in diameter) found in the sciatic MN pool that were cut in the nuclear plane were identified as alpha MNs by the presence of C-type nerve terminals. The terminals were categorized under high magnification as *F* (with flattened synaptic vesicles, inhibitory inputs), *S* (with spherical synaptic vesicles, excitatory glutamatergic inputs), or *C* (cholinergic inputs), according to the nomenclature of Conradi^64^.

The surface of the cells was then sequentially digitalized at a magnification of 13,000 ×. CorelDRAW (2018, Corel Corporation, Ottawa, CA) was then used to create a montage of the entire plasma membrane. The measurement tool of the ImageJ software (version 1.51J8, NIH, USA) was used to measure the total perimeter of the neuron and the apposed terminals. The number and length of synaptic terminals apposing the MN somata was obtained, and reported as the number of synaptic terminals per 100 μm of cell membrane.

### Statistics

Data were graphed and analyzed with Prism (GraphPad Software, Inc., San Diego, CA). One-way analysis of variance was performed with multiple groups. T-test was performed between only two groups. Linear regression was used for correlation analyses.

## Supplementary information

Supplemental data

Video

## Data Availability

The data are available from the corresponding author on reasonable request. All resources are available upon request from the corresponding author or from the stated commercial suppliers.
