# Supplementary material for: Zika virus infection causes temporary paralysis in adult mice with motor neuron synaptic retraction and evidence for proximal peripheral neuropathy
Source: Sci Rep. 2019 Dec 20;9:19531. doi: 10.1038/s41598-019-55717-3 (PMC6925114; doi:10.1038/s41598-019-55717-3)

**Supplementary information**

Zika virus infection causes temporary paralysis in adult mice with motor neuron synaptic retraction and evidence for proximal peripheral neuropathy

John D. Morrey^1^*^¶^, Alexandre L. R. Oliveira^2¶^, Hong Wang^1&^, Katherine Zukor^1&^, Mateus Vidigal de Castro^2&^, Venkatraman Siddharthan^1^

^1^Institute for Antiviral Research, Department of Animal, Dairy, and Veterinary Sciences, 5600 Old Main Hill, Utah State University, Logan, Utah, 84322-5600, United States of America

^2^Institute of Biology, University of Campinas, Campinas, SP, Brazil

**Figure S1.** Motor deficits and acute flaccid paralysis in ZIKV-infected 4-month-old *IFNAR^-/-^* mice monitored through day 51 after viral challenge. (**A,B,D,E**) VPS was measured in mice infected subcutaneously with 6.7 x 10^3^ pfu. (**A,B**) ZIKV-infected mice and (**C,D**) sham-infected mice.


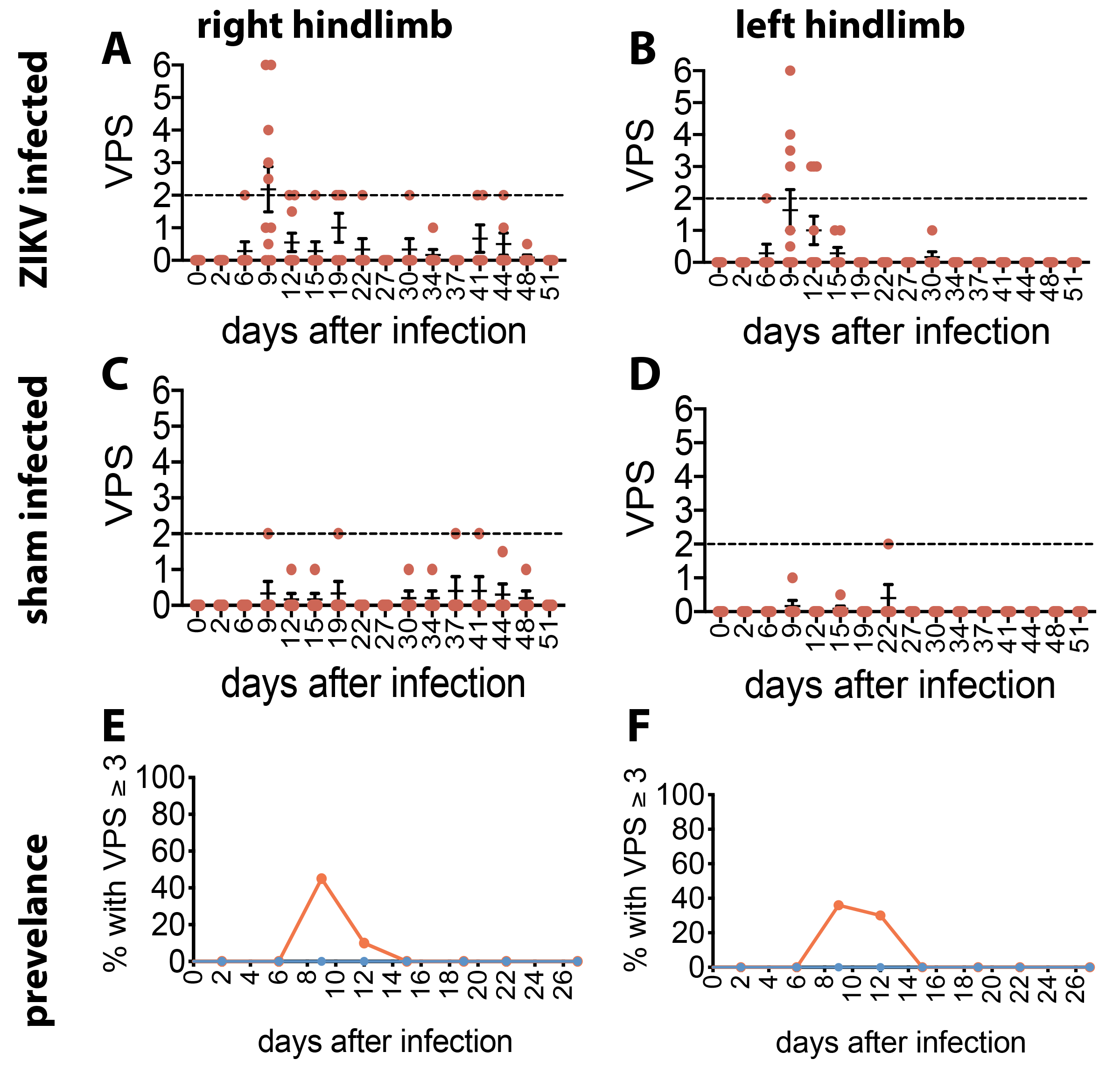


**Figure S2**. ZIKV challenge dose did not affect incidence of motor deficits in male and female *IFNAR^-/-^* mice, ages 3.6 – 4.0 months old, infected with 6.7 X 10^4^ pfu, 2.0 X 10^4^ pfu, or 6.7 X 10^3^ pfu per mouse of ZIKV. (**A,B**) 6.7 X 10^4^ pfu-group (n = 8 females and 4 males). (**C,D**) 2.0 X 10^4^ pfu-group (n = 8 females and 4 males). (**E,F**) 6.7 X 10^3^ pfu-group (n = 7 females and 5 males). Prevalence motor deficits identified as percent of VPS > 3 on the (**G**) right hindlimb and (**H**) left hindlimb. Hanging wire results on mice infected with (**I**) 6.7 X 10^4^ pfu, (**J**) 2.0 X 10^4^ pfu, or (**K**) 6.7 X 10^3^ pfu per mouse of ZIKV.

**
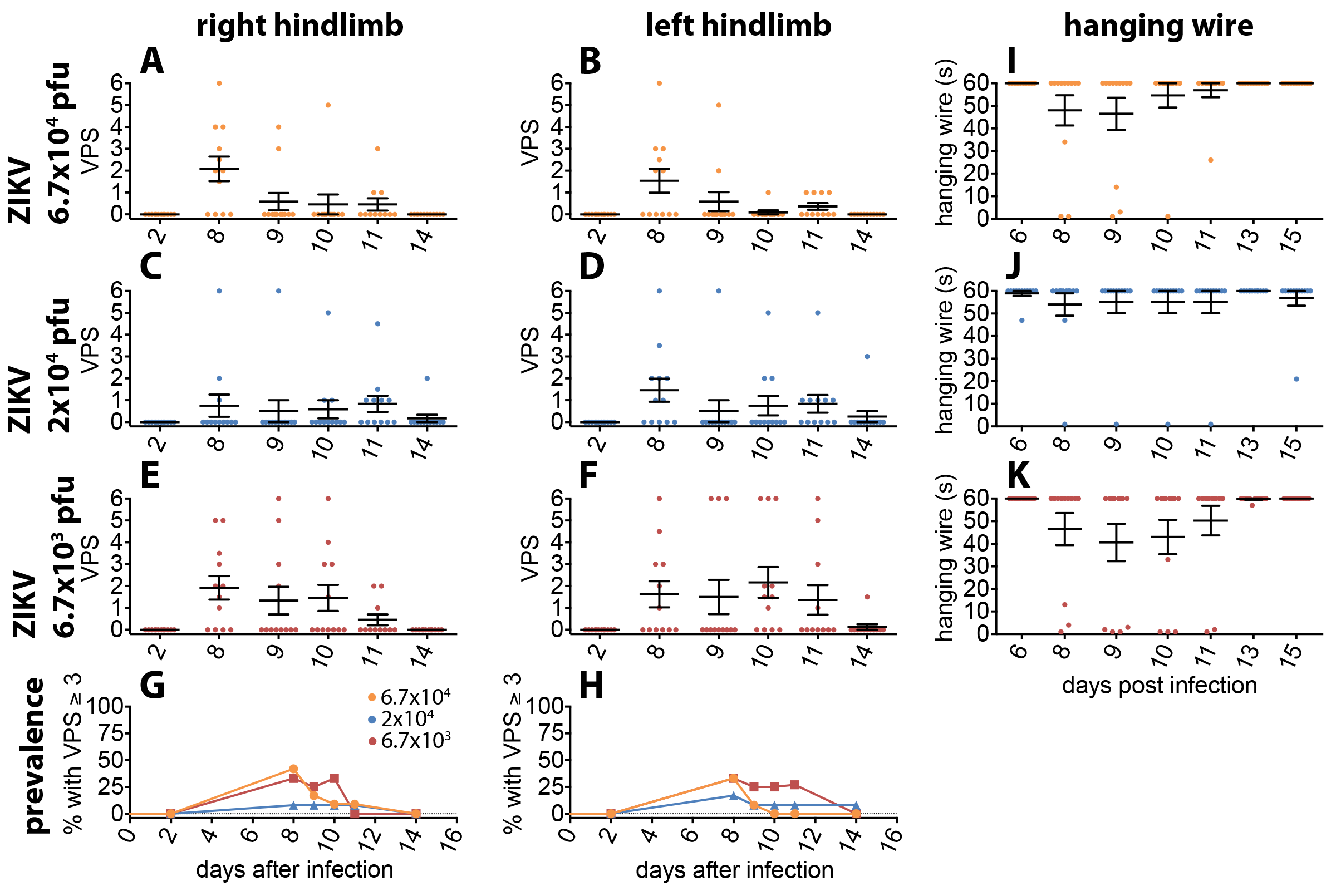
**

**Figure S3**. Hanging wire test correlates with VPS test. Male (n=17) and female (n=16) *IFNAR^-/-^* mice, ages 5.0 – 5.3 months old, were infected with 6.7 x 10^3^ pfu of ZIKV. Five animals (n=3 male, 2 female) received a sham infection, and 2 uninfected animals (females) received a sciatic nerve transection on the right hindlimb. The times of the hanging wire test was (**A**) 60 s and (**B**) 180 s. sum of R/L VPS = sum of right and left hindlimb VPS.

**Figure S4**. Paralysis is not caused by death of motor neurons (MNs). Animals that underwent electrophysiological analysis (Fig. 4) were perfused for immunohistochemical analysis at the end of the day of recording. Thus, the animals that underwent recording in the morning were perfused several hours after recording whereas animals that underwent recording in the afternoon were perfused soon after recording. (**A,D,G,J**) sham-infected mouse, (**B,E,H,K**) ZIKV-infected, paralyzed mouse, (**C,F,I,L**) ZIKV-infected, recovered mouse. (**A,B,C**) merged images of ChAT-ir, GFAP-ir, and DAPI and corresponding (**D,E,F**) ChAT-ir, (**G,H,I**) GFAP ir, (**J,K,L**) ZIKV-ir. Quantification of (**M**) numbers of MNs, (**N**) GFAP ir, (**O**) ZIKV ir. All *IFNAR^-/-^* mice were males (4.0 – 4.2 months old). One-way analysis was performed.

**
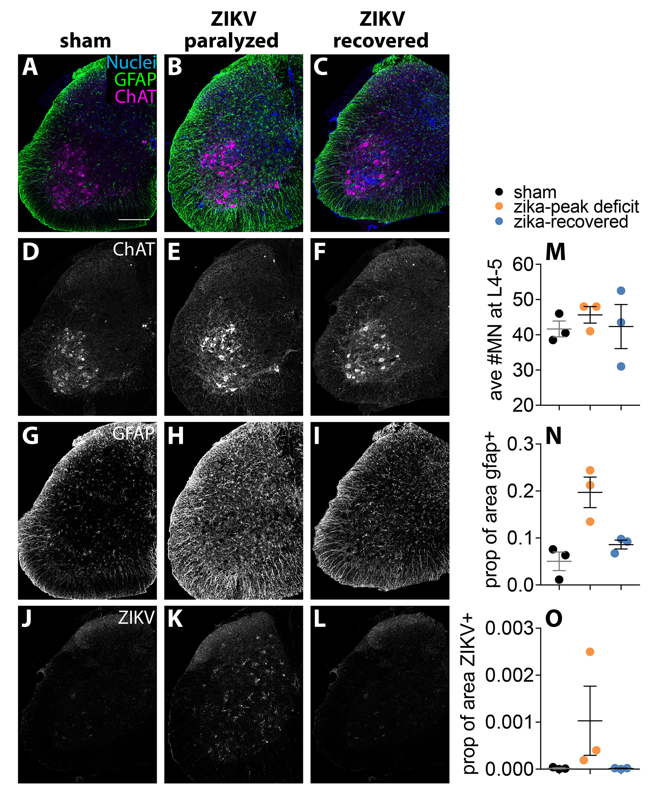
**

**Figure S5**. Inflammatory cells are present in the spinal cord of paralyzed animals. IHC ir of (**A,D,G,J**) sham-infected mouse, (**B,E,H,K**) ZIKV-infected, paralyzed mouse, (**C,F,I,L**) ZIKV-infected, recovered mouse. (**A,B,C**) merged images of CD3 ir, iba1 ir, and DAPI, (**D,E,F**) iba1 ir, (**G,H,I**) CD3 ir, (**J,K,L**) Ly6G ir. Quantification of (**M**) iba1 ir, (**N**) CD3 ir. (**O,P**) Iba1 ir in sham-infected and ZIKV-paralyzed mice showing amoeboid morphology. (**Q**) Ly6G, iba1, and CD3 ir in positive-control spleen and (**R**) spinal cord of a paralyzed mouse. N = 3 for all groups. One-way analysis was performed.

**
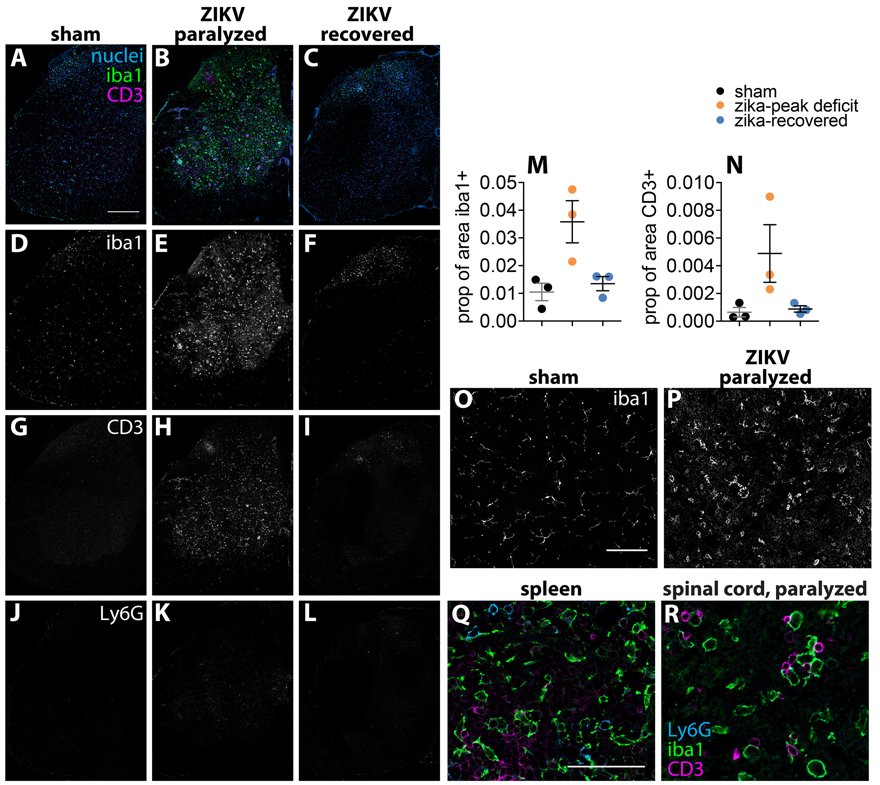
**

**Figure S6**. Neurofilament- and myelin basic protein-structures of the sciatic nerve are not structurally altered in the sciatic nerve of paralyzed mice. IHC ir of (**A,D,G**) sham-infected mouse, (**B,E,H**) ZIKV-infected, paralyzed mouse, (**C,F,I**) ZIKV-infected, recovered mouse. (**A,B,C**) merged images of neurofilament (NF) ir and myelin basic protein (MBP) ir, (**D,E,F**) ZIKV ir, (**G,H,I**) DAPI. Tyr = tyraminde enhancement. All *IFNAR^-/-^* mice were males (4.0 – 4.2 months old).

**
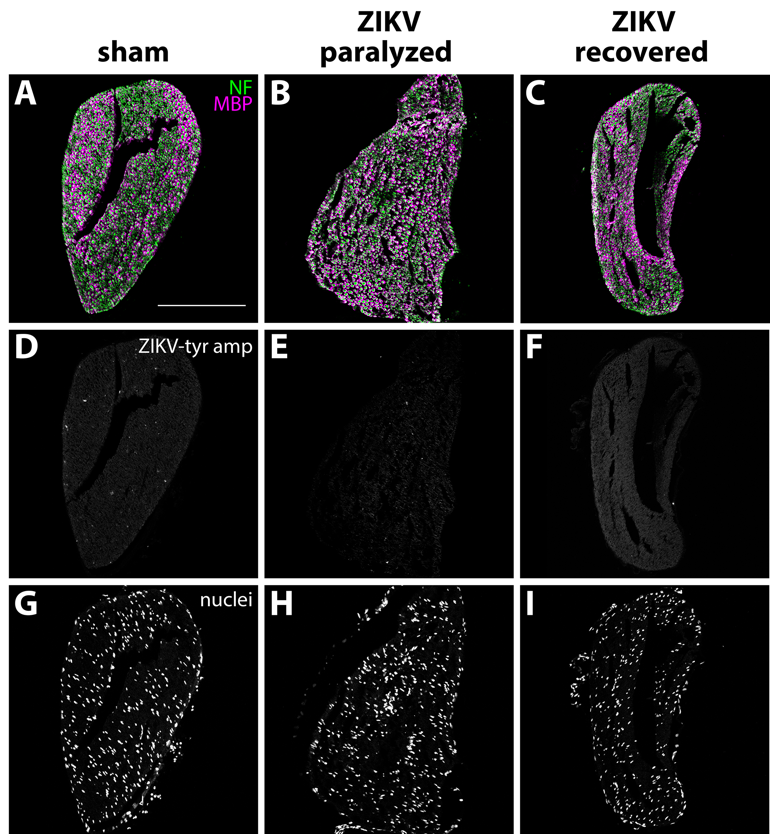
**

**Figure S7**. Macrophage infiltration into the sciatic nerve at the time of paralysis. Representative confocal images of the sciatic nerve of a (**A**) sham, (**B**) a paralyzed, ZIKV-infected animal (VPS right/left = 6/6), and (**C**) a recovering (after VPS right/left = 2.5/3) ZIKV-infected animal stained with iba1 (macrophages, green) and Hoechst (nuclei, blue). Quantification of the (**D**) proportion of the nerve occupied by iba1 positive pixels, or (**E**) cross-sectional stained area of the nerve in each group. Each dot represents one sciatic nerve. Bars represent the mean and error bars represent SEM. (**F-I**) Single section of control spleen tissue of (**F**) merge Ly6G (blue), iba1 (green), and CD3 (magenta) staining, and (**G**) iba1, (**H**) CD3, and (**I**) Ly6G individually. Scale bars: 250 μm in A (A-C same scale), 100 μm in F (F-I same scale). One-way analysis was performed.


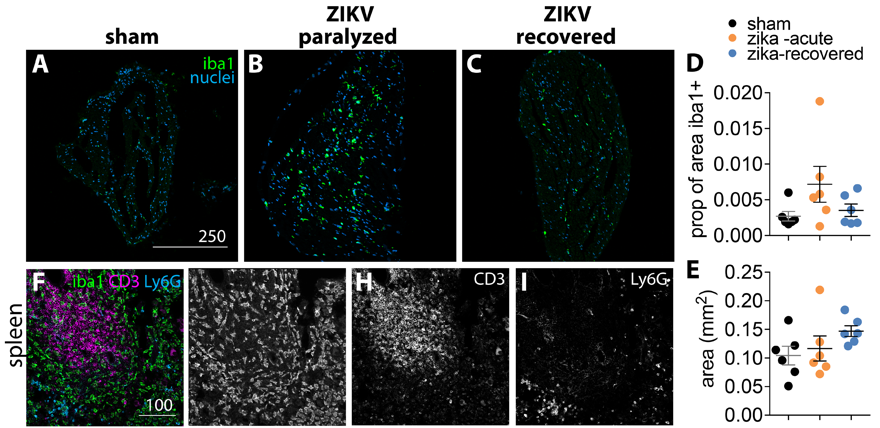


**Figure S8.** F-wave latency increased and % F-wave decreased in ZIKV-infected mice. (**A**) F-wave persistence, (**B**) % F-wave latency, (**C**) F-wave amplitude. Ages 16 to 17-week-old male and female mice were infected with ZIKV or sham. The F-wave assay was performed on three ZIKV-infected mice (males) with VPS = 5 or 6 on days 9 and 10. Two male and three female sham-infected mice were assayed on days 8 and 11, respectively. Sham n = 5; ZIKV n = 4. Data points represent individual hindlimbs. T-test was performed.


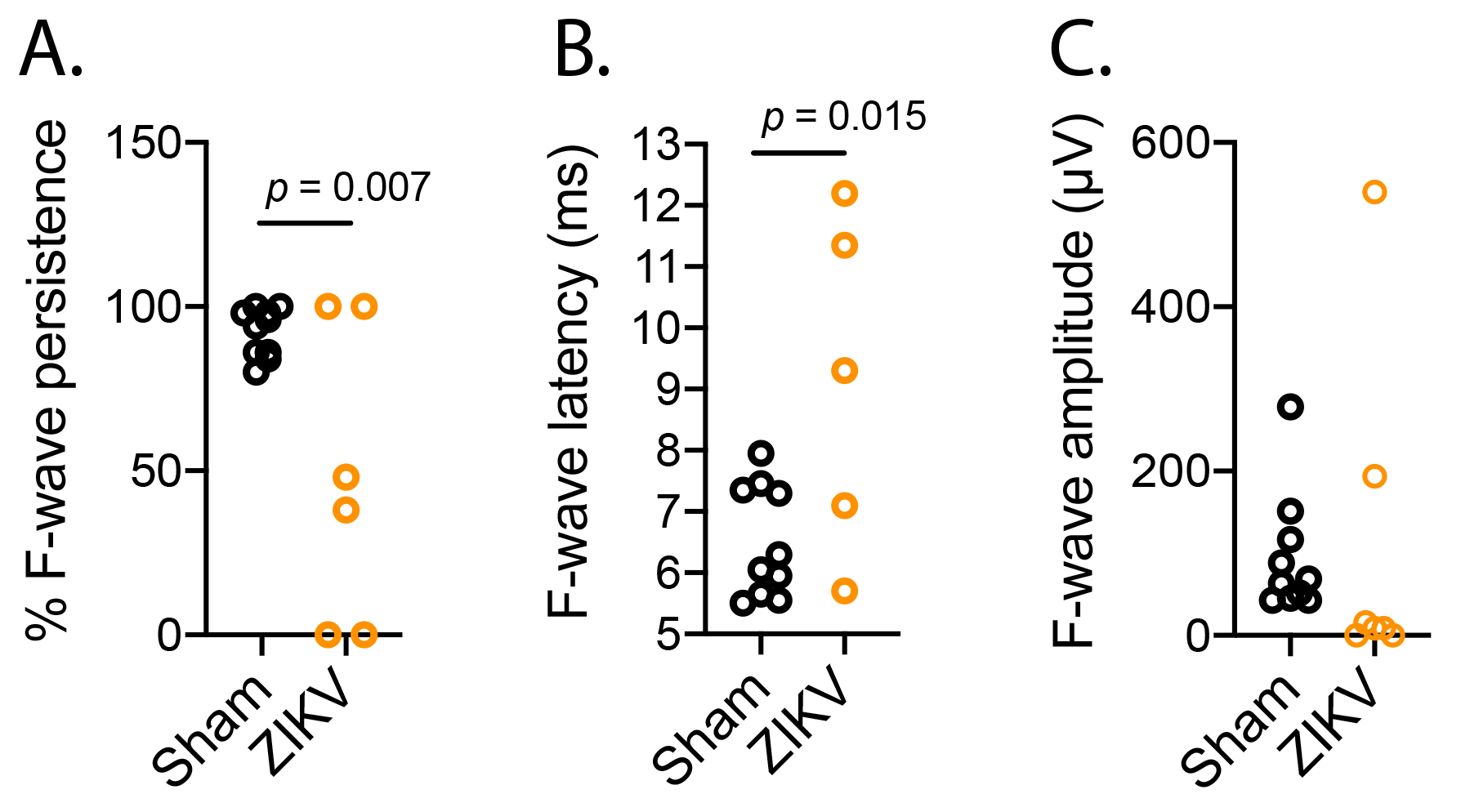

Supplement: Supplementary file 1 — Supplemental data [file 41598_2019_55717_MOESM1_ESM.docx]
